# Author Correction: *In operando* NMR investigations of the aqueous electrolyte chemistry during electrolytic CO_2_ reduction

**DOI:** 10.1038/s42004-024-01156-9

**Published:** 2024-04-01

**Authors:** Sven Jovanovic, Peter Jakes, Steffen Merz, Davis Thomas Daniel, Rüdiger-A. Eichel, Josef Granwehr

**Affiliations:** 1 Institute of Energy and Climate Research - Fundamental Electrochemistry (IEK-9), Forschungszentrum Jülich GmbH, Willhelm-Johnen-Straße, Jülich, Germany; 2 Institute of Physical Chemistry (IPC), RWTH Aachen University, Aachen, Germany; 3 Institute of Technical and Macromolecular Chemistry (ITMC), RWTH Aachen University, Aachen, Germany

**Keywords:** Electrocatalysis, Solution-state NMR, Electrocatalysis

Correction to: *Communications Chemistry* 10.1038/s42004-023-01065-3, published online 6 December 2023

The original version of this Article contained errors in the Data availability statement, which incorrectly read ‘All data reported in this work are available from the authors by request. Please refer to the corresponding author Sven Jovanovic (s.jovanovic@fz-juelich.de).’ The correct version states ‘NMR data are available for download at 10.26165/JUELICH-DATA/0GQJFC. Other data are available from the authors upon request.’ This has now been corrected in both the PDF and HTML versions of the Article.

