# Supplementary material for: In operando NMR investigations of the aqueous electrolyte chemistry during electrolytic CO2 reduction
Source: Commun Chem. 2023 Dec 6;6:268. doi: 10.1038/s42004-023-01065-3 (PMC10700511; doi:10.1038/s42004-023-01065-3)
Supplement: Supplementary file 2 — Supplementary Information [file 42004_2023_1065_MOESM2_ESM.pdf]

Supplementary Information

Sven Jovanovic[1,*], Peter Jakes[1], Steffen Merz[1], Davis Thomas Daniel[1], Rüdiger-A. Eichel[1,2], and Josef Granwehr[1,3]

[1]Institute of Energy and Climate Research - Fundamental Electrochemistry (IEK-9), Forschungszentrum Jülich GmbH, Willhelm-Johnen-Straße, 52428 Jülich, Germany
[2]Institute of Physical Chemistry (IPC), RWTH Aachen University, Landoltweg 2, 52074 Aachen, Germany
[3]Institute of Technical and Macromolecular Chemistry (ITMC), RWTH Aachen University, Worringerweg 1-2 , 52074 Aachen, Germany

Supplementary figures

In operando NMR spectra

^{13}C spectra at B_0 =14.1 T

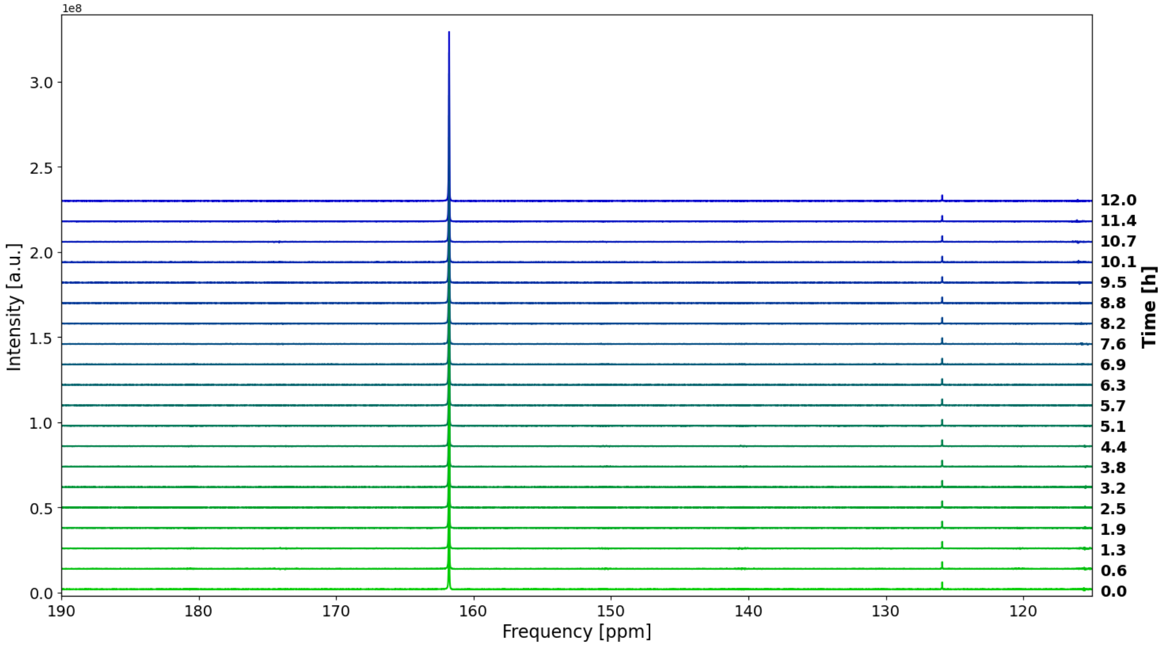

Figure S1. Time evolution of ^{13}C spectra recorded under OCV conditions and at B_0 =14.1 T. The features at ca. 115 ppm are due to the amplication of external radiofrequency noise by the electrolysis setup.

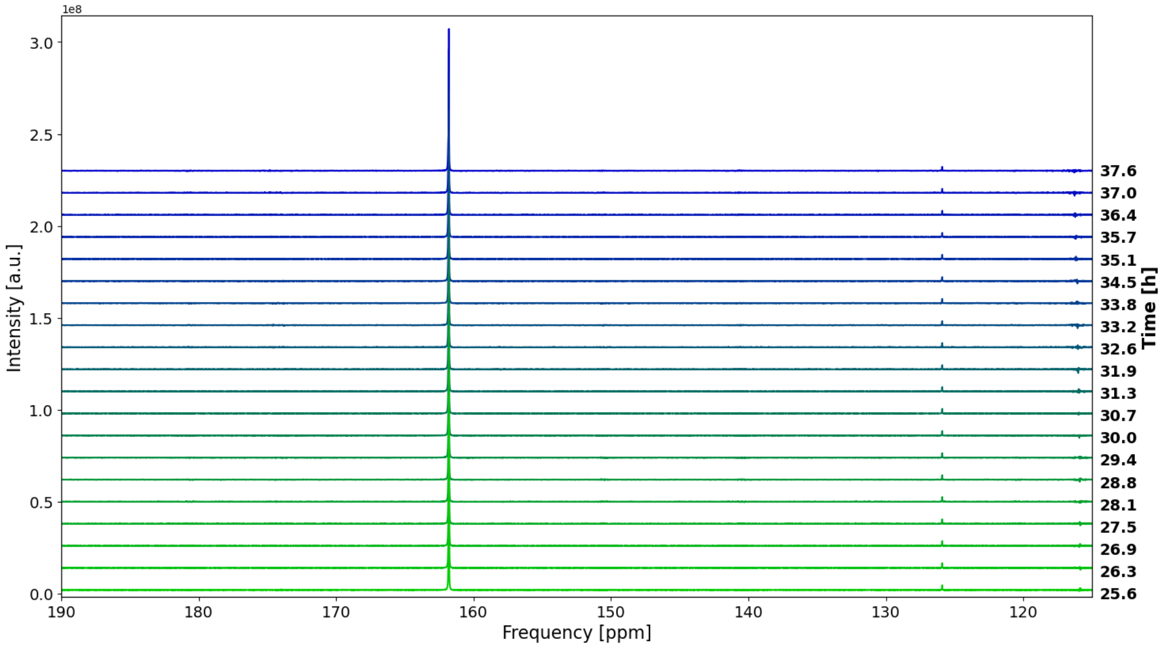

Figure S2. Time evolution of ^{13}C spectra recorded under CA conditions and at B_0 =14.1 T. TThe features at ca. 115 ppm are due to the amplication of external radiofrequency noise by the electrolysis setup.

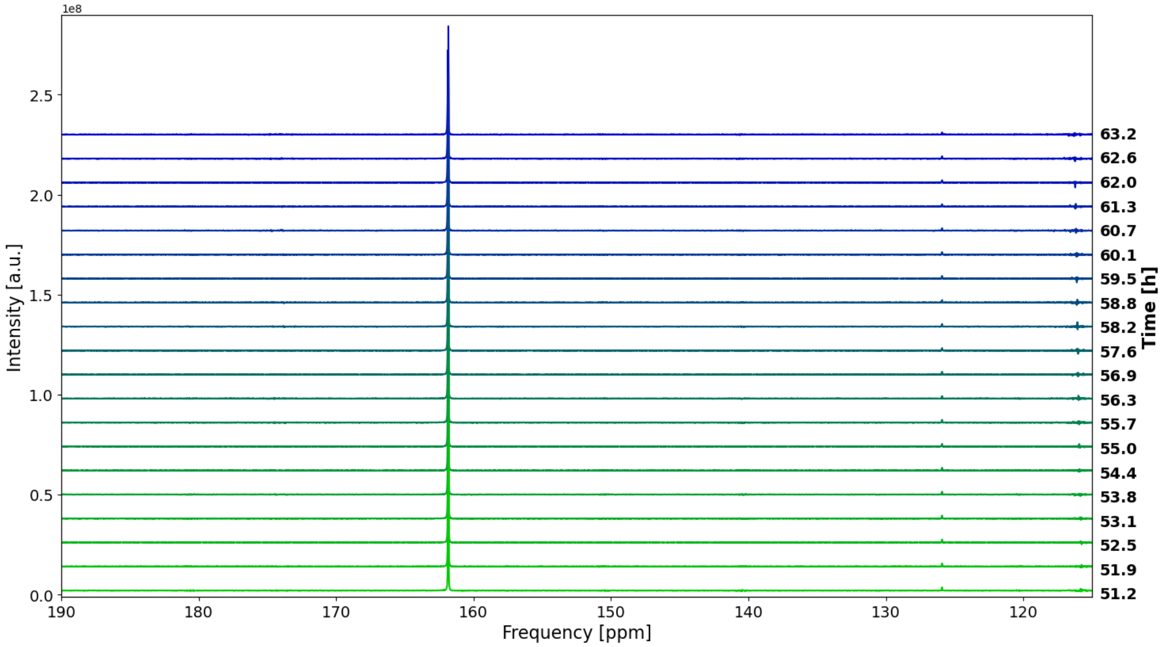

Figure S3. Time evolution of ^{13}C spectra recorded at CP conditions and at B_0 =14.1 T. TThe features at ca. 115 ppm are due to the amplication of external radiofrequency noise by the electrolysis setup.

^{13}C spectra at B_0 =9.4 T

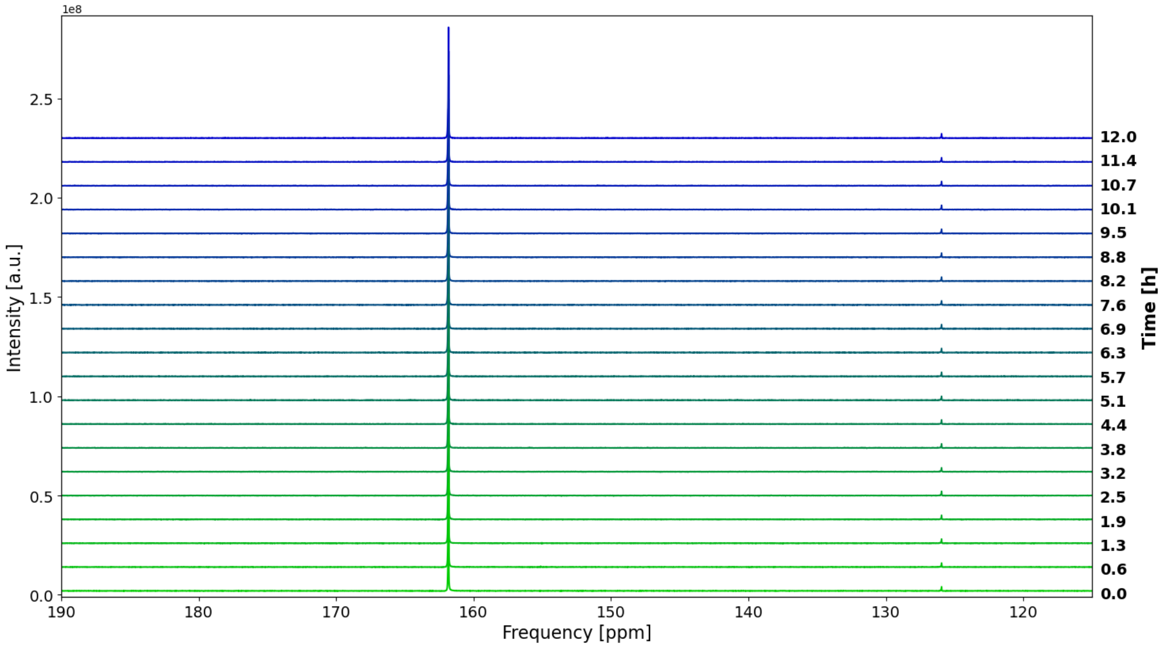

Figure S4. Time evolution of ^{13}C spectra recorded under OCV conditions and at B_0 =9.4 T.

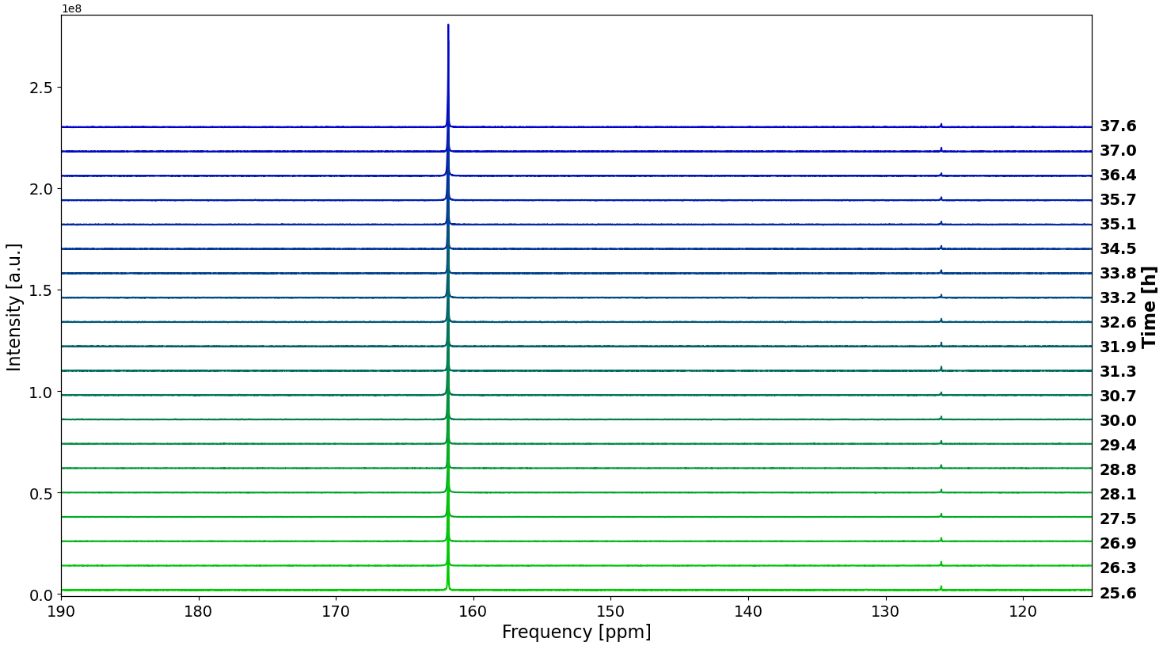

Figure S5. Time evolution of ^{13}C spectra recorded under CA conditions and at B_0 =9.4 T.

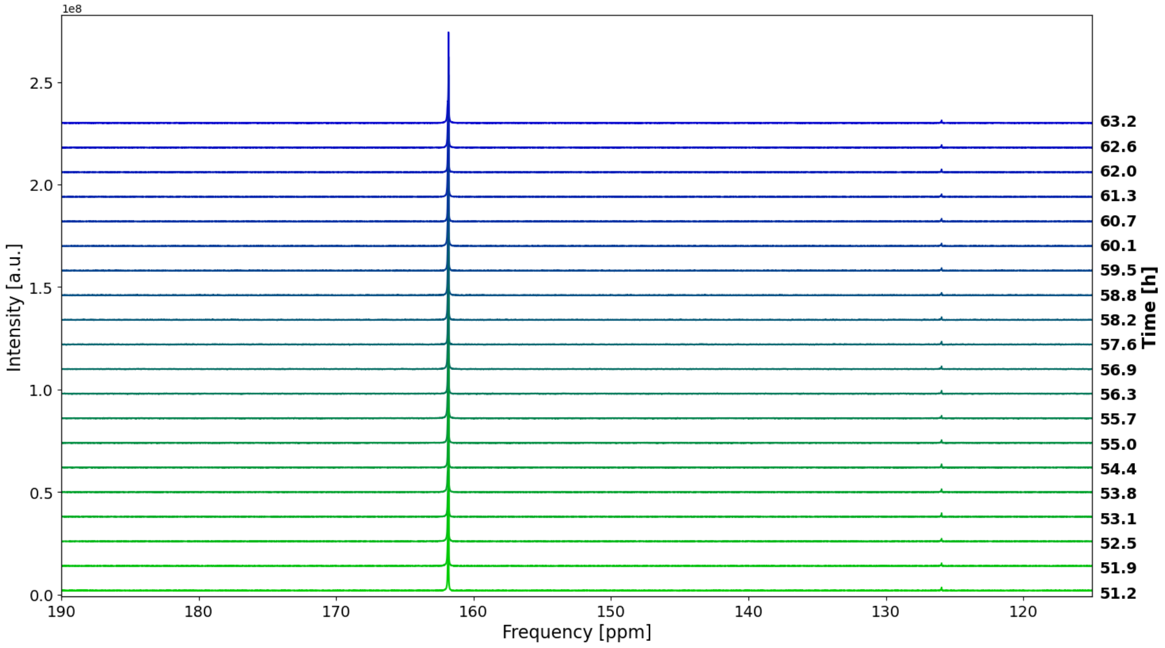

Figure S6. Time evolution of ^{13}C spectra recorded under CP conditions and at B_0 =9.4 T.

^{23}Na spectra at B_0 =14.1 T

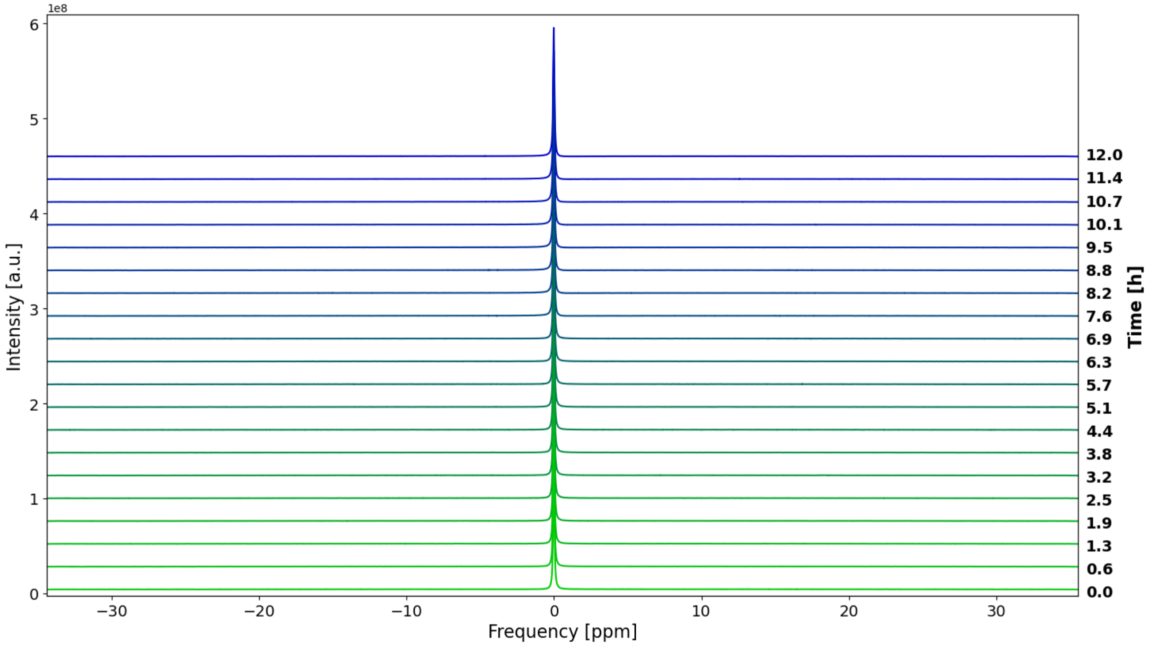

Figure S7. Time evolution of ^{23}Na spectra recorded under OCV conditions and at B_0 =14.1 T.

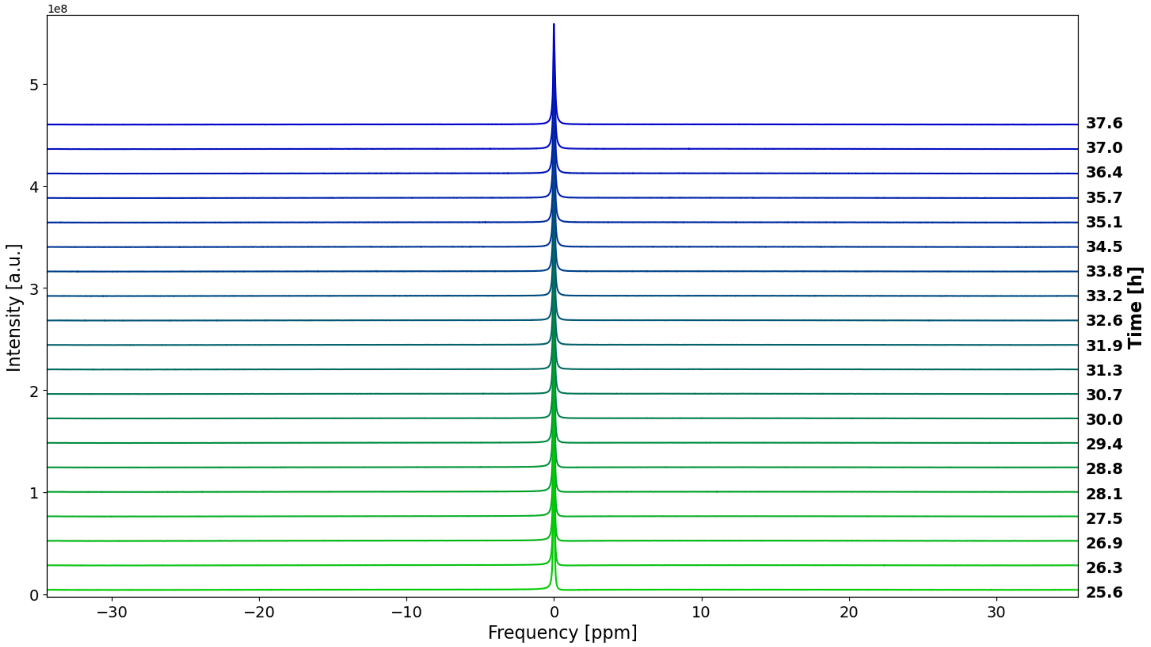

Figure S8. Time evolution of ^{23}Na spectra recorded under CA conditions and at B_0 =14.1 T.

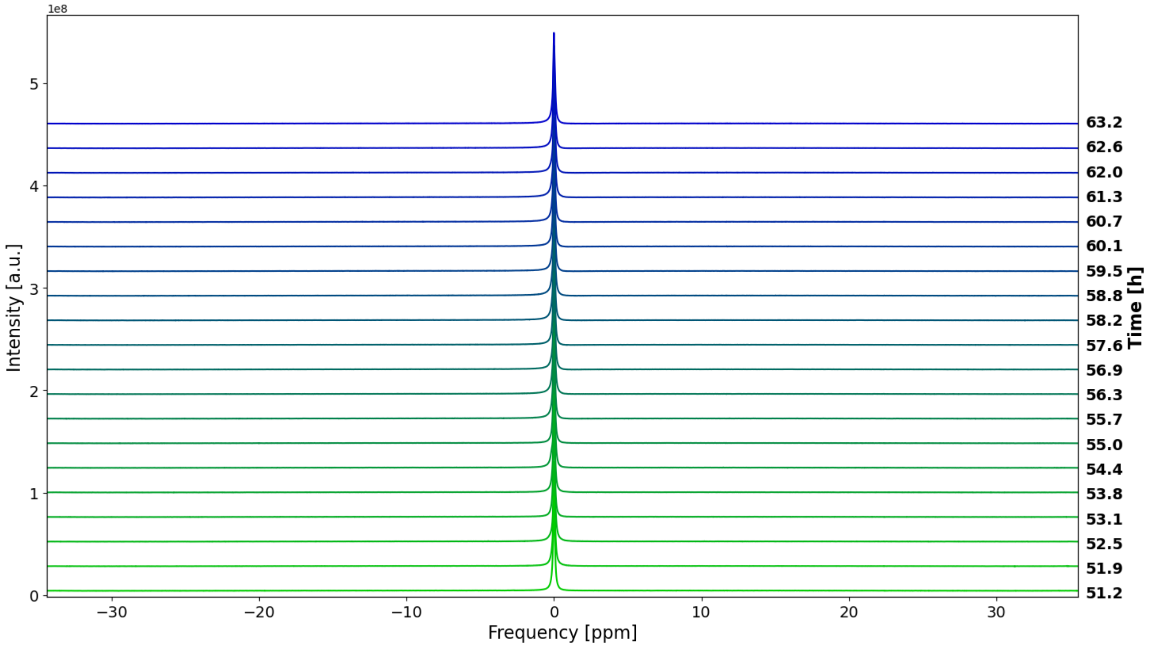

Figure S9. Time evolution of ^{23}Na spectra recorded under CP conditions and at B_0 =14.1 T.

Evaluation of T_1, T_2 and EXSY NMR experiments at 14.1 T

T_1 determination by saturation recovery experiments

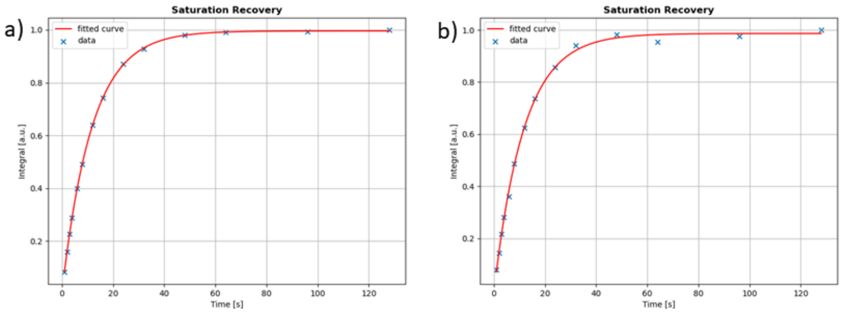

Figure S10. Evaluation of the saturation recovery experiment during the OCV stage at 14.1 T for the individual $^{13}C\ HCO_3^-$ signal components: a) Coalesced signal b) B_0 distortion. T_1 was determined by fitting a monoexponentiell function to the time evolution of the signal integral.

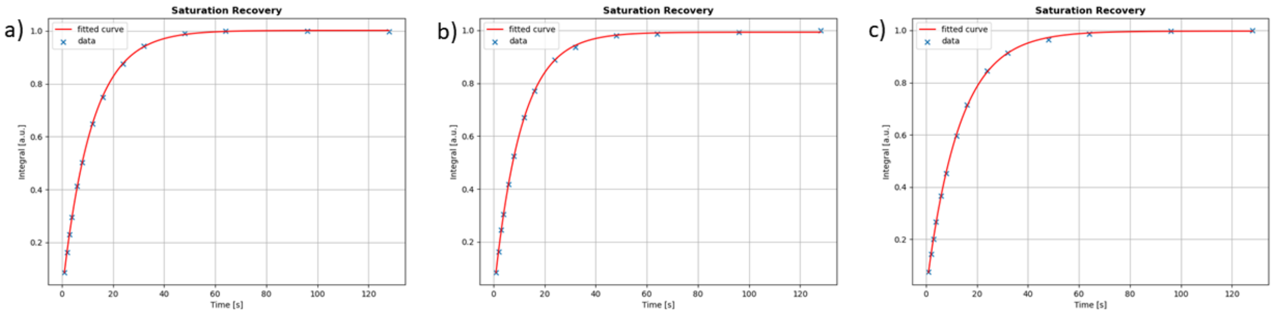

Figure S11. Evaluation of the saturation recovery experiment during the CA stage for the individual $^{13}C\ HCO_3^-$ signal components: a) Free ions, b) Ion pairs, c) B_0 distortion. T_1 was determined by fitting a monoexponentiell function to the time evolution of the signal integral.

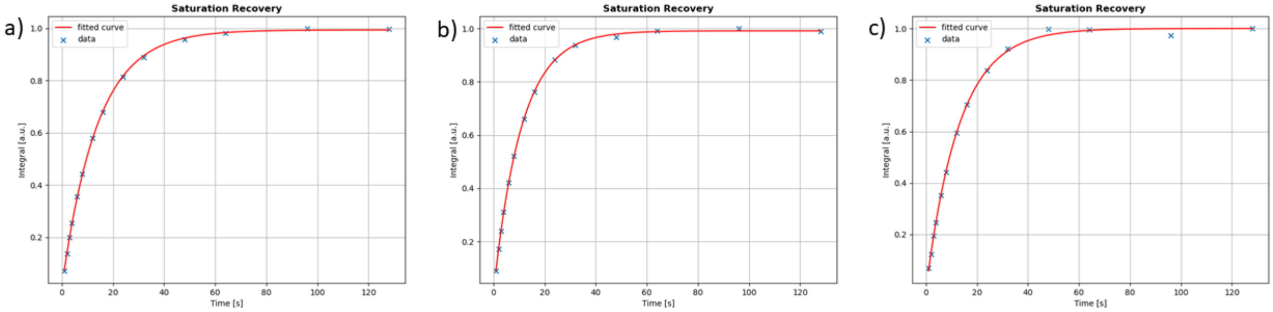

Figure S12. Evaluation of the saturation recovery experiment at 14.1 T during the CP stage for the individual $^{13}C\ HCO_3{}^-$ signal components: a) Free ions, b) Ion pairs, c) B_0 distortion. T_1 was determined by fitting a monoexponentiell function to the time evolution of the signal integral.

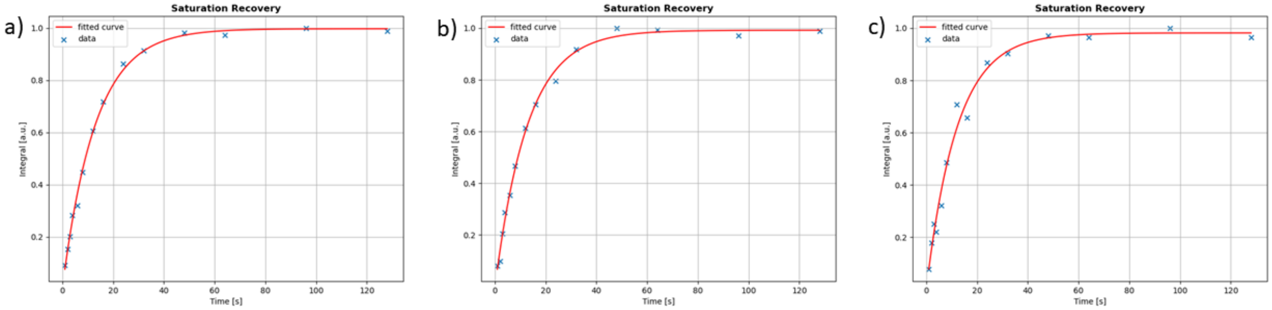

Figure S13. Evaluation of the saturation recovery experiment at 14.1 T for the $^{13}C\ CO_2$ signal at the three different experimental stages: a) OCV, b) CA, c) CP. T_1 was determined by fitting a monoexponentiell function to the time evolution of the CO_2 signal integral.

T_2 ***determination by CPMG experiments***

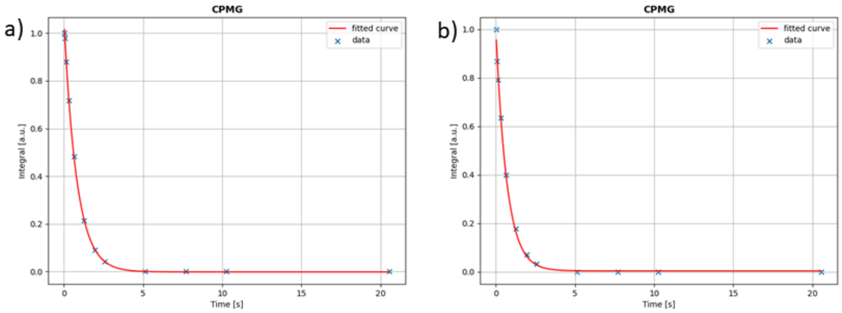

Figure S14. Evaluation of the CPMG experiment at 14.1 T during the OCV stage for the individual ^{13}C HCO_3^- signal components: a) Coalesced signal b) B_0 distortion. T_1 was determined by fitting a monoexponentiell function to the time evolution of the signal integral.

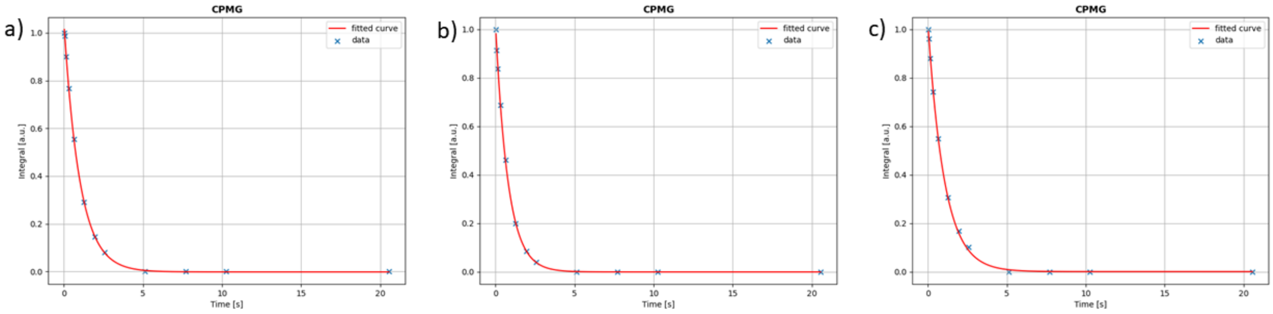

Figure S15. Evaluation of the CPMG experiment at 14.1 T during the CA stage for the individual ^{13}C HCO_3^- signal components: a) Free ions, b) Ion pairs, c) B_0 distortion. T_1 was determined by fitting a monoexponentiell function to the time evolution of the signal integral.

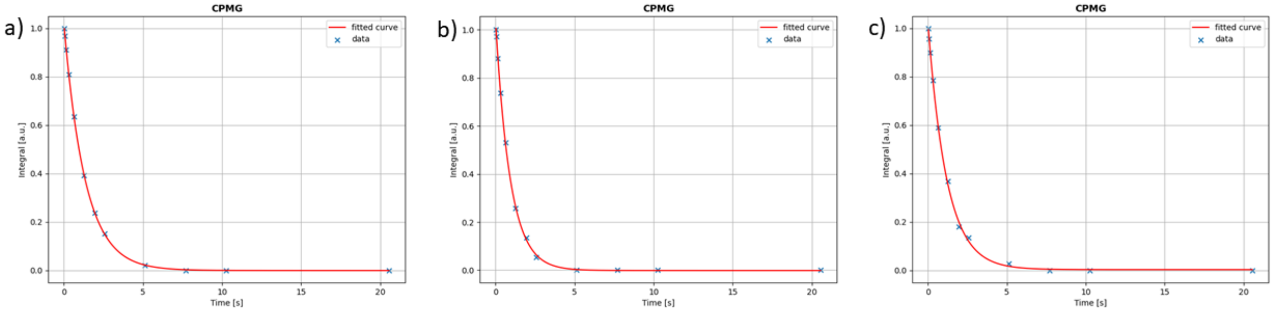

Figure S16. Evaluation of the CPMG experiment at 14.1 T during the CP stage for the individual ^{13}C HCO_3^- signal components: a) Free ions, b) Ion pairs, c) B_0 distortion. T_1 was determined by fitting a monoexponentiell function to the time evolution of the signal integral.

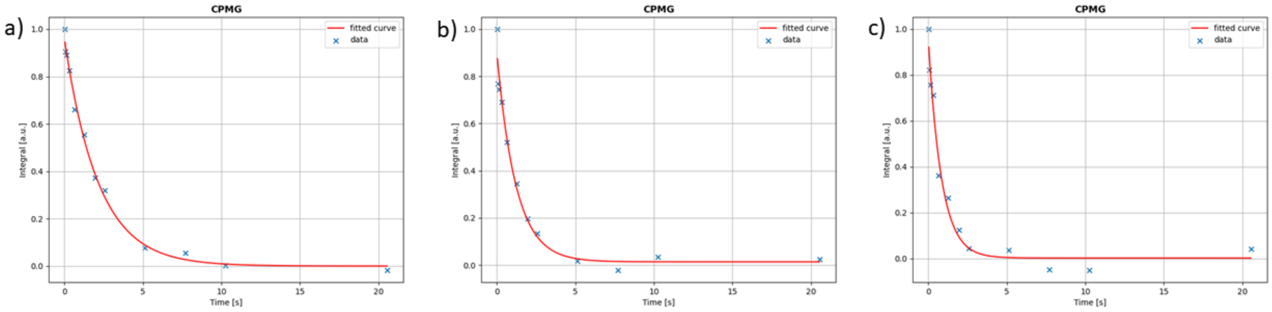

Figure S17. Evaluation of the CPMG experiment at 14.1 T for the ^{13}C CO_2 signal at the three different experimental stages: a) OCV, b) CA, c) CP. T_2 was determined by fitting a monoexponentiell function to the time evolution of the CO_2 signal integral.

EXSY experiments

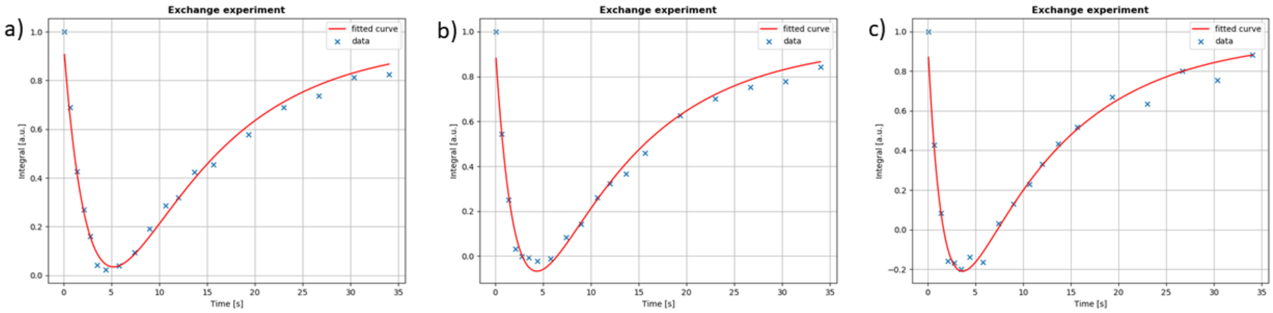

Figure S18. Evaluation of the EXSY experiment at 14.1 T during the OCV stage at the three different experimental stages: a) OCV, b) CA, c) CP. T_{exc} was determined by fitting eq. 14 given in the main text to the time evolution of the CO_2 signal integral.

Supplementary Methods

DFT Chemical shift simulations

Static hydration sphere model

A contact pair of sodium and bicarbonate ions with with 5 water molecules per ion[1,2] was geometry optimized to generate the first structure for the static hydration sphere model. The geometry and alignment of the solvation shells was then left static, and only the Na–O distance was varied to generate structures with Na-O distances ranging from 2.8 Å to 9.5 Å. Figure S19 depicts an example geometry at a distance of 3.6 Å. The ^{13}C and ^{23}Na chemical shifts were calculated for these structures and are dipicted in fig. S20. As no further changes of the chemical shifts were observed for Na–O distances greater than 9.5 Å, these structures were considered as free ions.

The chemical shift is strongly affected for structures with a Na–O distance smaller than 3.5 Å, *i.e.* for contact ion pairs, but the effect quickly drops off for larger distances. The region between 3.5 and 4 Å (shaded) exhibits a chemical shift range which is in agreement with experimental data, and corresponds to the distances expected for SIPs. For distances up to 4 Å the ion pair is shifted downfield compared to the free ions. In case of ^{13}C chemical shifts, this is expected as electron density from the anion is transferred to the anion to the cation. However, for even higher distances the ion pairs signals are shifted upfield instead. It is unclear whether this behavior is an artefact of the simple model used for this study. At Na–O distances up 8–9 Å, the interaction between the ions is weakened and the chemical shift difference approaches zero.

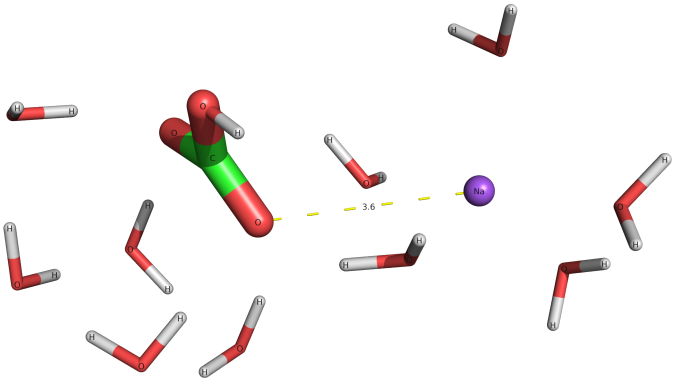

Figure S19. Geometry optimized Na^+ HCO_3^- ion pair with static hydration spheres at a Na-O distance of 3.6 Å.

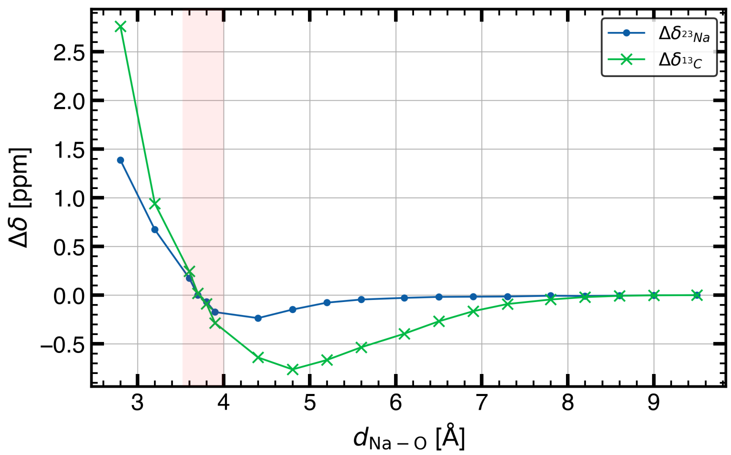

Figure S20. Evolution of the ^{13}C and ^{23}Na chemical shift differences between ion pairs and free ions. The chemical shift of the ion pair configuration corresponding to a Na-O distance of 9.5 Å is considered as the chemical shift of free ions.

Molecular Dynamics model

For the ab-initio molecular dynamics simulations, the evolution of the hydration sphere of a $Na^{+}HCO_3^{-}$ ion pair was simulated over the course of 1000 fs at four distances (3.3, 4.0, 7.5, 9.2 Å), representing CP, SIP, 2SIP and free ions. As for the static model, 5 water molecules per ion were used. Every 5 fs, a snapshot of the geometry was taken and used for DFT chemical shift calculations. The resulting evolution of ^{13}C and ^{23}Na calculations over the course of the simulated time frame is depicted in fig. S21. In case of the bicarbonate anion, *i.e.* for the ^{13}C shifts, sufficient averaging of the hydration sphere geometry was observed over 1000 fs. The sodium cation hydration sphere is evolving significantly slower. Thus, a higher error is expected for the ^{23}Na chemical shift calculations. Longer MD simulation times were not feasible due to high computational costs.

The averaged chemical shifts, depicted as differences between ion pairs and the free ion at 9.2 Å, are shown in table S1. For ^{13}C, the chemical shift differences are in good agreement with the experiments for ion distances of 4.0 Å and 9.2 Å. Moreover, the calculated ion pair signals are shifted downfield. In case of ^{23}Na chemical shifts, simulations do not match the experimental data as well as well, but are on the same order of magnitude, and the ion pair signal is also shifted downfield. This may be due to the worse averaging observed for the hydration geometry of the sodium cation.

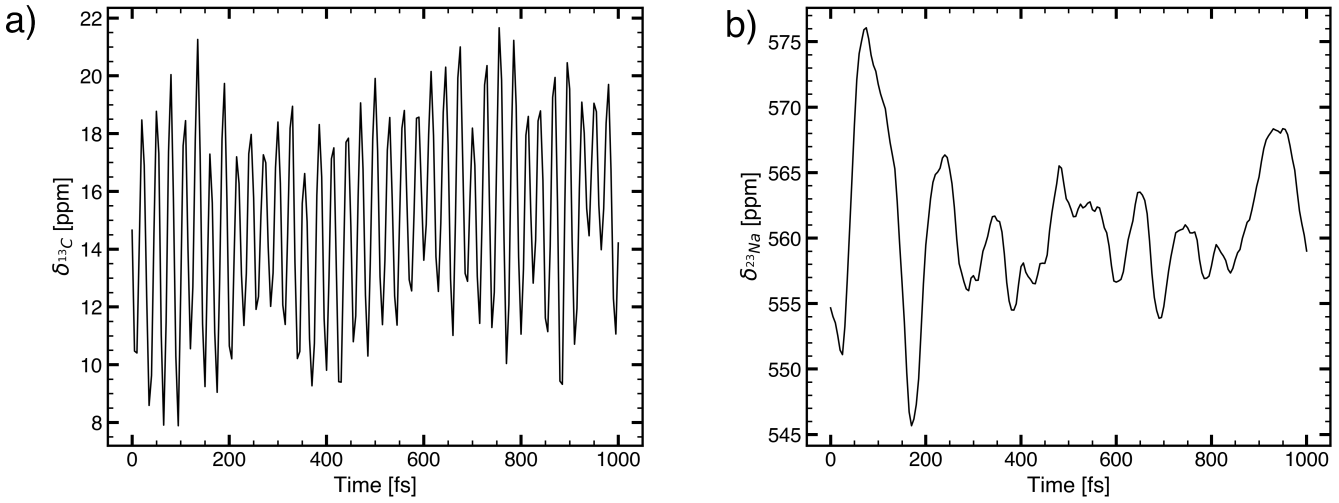

Figure S21. ^{13}C (a) and ^{23}Na (b) chemical shift calculations for MD snapshots of the ion pair system with a Na-C distance constraint of 9.2 Å applied during the MD run. Note that the chemical shift is not referenced, and thus the absolute values are arbitrary. For ^{13}C, the chosen time frame of 1 ps is sufficient to average over a representative number of hydration sphere geometries. In case of ^{23}Na, the change in hydration sphere geometry evolves significantly slower.

Table S1. Average chemical shift differences between free ions and ion pairs obtained by chemical shift calculations using distance constrained ion pair structures extracted from MD simulations.

Na-C distance [Å]	$\Delta\delta_{^{13}C}$ [ppm]	$\Delta\delta_{^{23}Na}$ [ppm]
3.3	2.0	2.3
4.0	0.9	1.1
7.5	0.4	2.5
9.2	0.0	0.0

References

1. M.A., T., E.A., G. & M.H.A., S. Aqueous micro-hydration of na+(h2o)n=1-7 clusters: Dft study. *Open Chem.* **17**, 260–269, DOI: doi:10.1515/chem-2019-0025 (2019).

2. Dopieralski, P. D., Burakowski, A., Latajka, Z. & Olovsson, I. Hydration of nahco3, khco3, (hco3-)2, hco3- and co32- from molecular dynamics simulation and speed of sound measurements. *Chem. Phys. Lett.* **507**, 89–95, DOI: https://doi.org/10.1016/j.cplett.2011.03.065 (2011).
